# Error Awareness and Salience Processing in the Oddball Task: Shared Neural Mechanisms

**DOI:** 10.3389/fnhum.2012.00246

**Published:** 2012-08-27

**Authors:** Helga A. Harsay, Marcus Spaan, Jasper G. Wijnen, K. Richard Ridderinkhof

**Affiliations:** ^1^Department of Psychology, Amsterdam Center for the Study of Adaptive Control in Brain and Behavior, University of AmsterdamAmsterdam, Netherlands; ^2^Cognitive Science Center Amsterdam, University of AmsterdamAmsterdam, Netherlands

**Keywords:** anterior insula, error awareness, oddball processing, salience, magnetic resonance imaging, eyetracking

## Abstract

A body of work suggests similarities in the way we become aware of an error and process motivationally salient events. Yet, evidence for a shared neural mechanism has not been provided. A within subject investigation of the brain regions involved in error awareness and salience processing has not been reported. While the neural response to motivationally salient events is classically studied during target detection after longer target-to-target intervals in an oddball task and engages a widespread insula-thalamo-cortical brain network, error awareness has recently been linked to, most prominently, anterior insula cortex. Here we explore whether the anterior insula activation for error awareness is related to salience processing, by testing for activation overlap in subjects undergoing two different task settings. Using a within subjects design, we show activation overlap in six major brain areas during aware errors in an antisaccade task and during target detection after longer target-to-target intervals in an oddball task: anterior insula, anterior cingulate, supplementary motor area, thalamus, brainstem, and parietal lobe. Within subject analyses shows that the insula is engaged in both error awareness and the processing of salience, and that the anterior insula is more involved in both processes than the posterior insula. The results of a fine-grained spatial pattern overlap analysis between active clusters in the same subjects indicates that even if the anterior insula is activated for both error awareness and salience processing, the two types of processes might tend to activate non-identical neural ensembles on a finer-grained spatial level. Together, these outcomes suggest a similar functional phenomenon in the two different task settings. Error awareness and salience processing share a functional anatomy, with a tendency toward subregional dorsal and ventral specialization within the anterior insula.

## Introduction

When we interact with our environment, neural activity enabling goal-directed behavior is formed and continuously updated in order to adjust new action based on the experience of previous actions. As human behavior is susceptible to occasional errors, the ability to become aware of such errors keeps us from repeating the inadequate actions. This protects us from potentially harmful situations. How the brain instigates the ability to become aware of errors is yet unknown. Initial neuroimaging evidence suggests that, while error processing *per se* engages both anterior insula cortex (AIC) and anterior cingulate cortex (ACC), error awareness engages only the AIC (Klein et al., 2007). The functional significance of this AIC activation is however unclear.

One potential way to further our understanding of error awareness has been suggested by event-related potential (ERP)-work. ERP-studies on error awareness suggest neural similarities in the way we become aware of an error and attend to salient events (Ridderinkhof et al., 2009). The ability to attend to salient events is a basic ability that helps us to attend to meaningful events that have motivational importance. This ability is typically studied in a classical oddball paradigm, requiring the detection of distinct infrequent target stimuli or oddballs which are embedded in a series of frequently presented non-target or standard stimuli (Duncan-Johnson et al., 1984). The neural circuits that mediate oddball processing are well delineated by ERP as well as neuroimaging work (Kiehl and Liddle, 2003; Kiehl et al., 2005; Stevens et al., 2005). Whether the similarity as present in ERP work is also apparent in the neuroimaging manifestations of error awareness and oddball processing is yet unknown.

Here we set out to provide a test of the hypothesis that the AIC engaged during error awareness is also recruited (in the same subjects during the same session) in an oddball task during manipulations of oddball stimuli known to affect the processing of motivational salience.

### The salience system

Generally, the insula is viewed as a dynamic interactive structure. It is well-placed to evaluate the motivational or emotional salience of certain events and is acting as an interface between external information and internal motivational states (Mesulam and Mufson, 1982a,b; Mufson and Mesulam, 1982; Craig, 2002, 2009; Seeley et al., 2007). Differences have been found in structural connectivity and in evoked responses to specific tasks across subregions of the insula (Dupont et al., 2003; Mutschler et al., 2009; Deen et al., 2011).

In order to appreciate the activation of the anterior subregion of the insula during error awareness, we adopt a systems perspective that considers complex and multi-faceted functions to arise from the dynamic interactions of larger scale brain systems connected to this anterior subregion (Bressler and Menon, 2010). This principled theoretical perspective may aptly guide our exploration of how activation in the AIC can promote as well as constrain the emergence of salience signaling in both error awareness and the parametrical oddball task.

The use of various neuroimaging techniques has helped characterize a number of large-scale brain systems. Such systems may be configured dynamically and transiently, in response to current task demands, whereas other systems may be more fundamental and constant, so as to deal consistently and generically with common or recurrent demands. One of these networks comprises the dorsal ACC and the AIC/frontal operculum, a consistently observed functional network, described as a salience or control network (Dosenbach et al., 2007, 2008). This AIC–ACC network was initially thought to be task-specific, involved in the initiation and maintenance of task set, in task control such as monitoring, error feedback, and in subsequent performance adjustments. When a similar AIC–ACC network was subsequently identified in task-free states, it became termed the salience network (Menon and Uddin, 2010), thought to be involved in orienting to homeostatically relevant (salient) intrapersonal and extrapersonal events. The AIC and ACC often act in concert, as supported by findings of reciprocal projections in monkeys. Resting-state fMRI studies also indicate functional connectivity between anterior insula and the ACC (Taylor et al., 2009).

Not surprisingly, then, the AIC and ACC are often found to be co-activated in functional neuroimaging studies, in particular in response to the degree of subjective salience across domains (Sridharan et al., 2008; Craig, 2009). Co-activation of these core components of the salience network has been associated with orienting to, and facilitating the processing of personally and motivationally salient information, in the broad spectrum of emotional, social, cognitive, sensorimotor, homeostatic, and sympathetic efferent and interoceptive autonomic domains. Within the salience network, the AIC appears more specialized in receiving multimodal sensory input, whereas the ACC is connected more to action selection and action execution systems in cortical and subcortical brain regions, allowing the salience network to influence not only attention (to facilitate the further processing of salient signals) but also adaptive action in response to such signals.

### The core function of the salience system

Identifying motivationally salient stimuli has been proposed as the core function of the salience system; once a stimulus activates the salience system, it will have preferential access to the brain’s attentional and working memory resources (Menon and Uddin, 2010). That is, once sensory areas detect a salient stimulus, this signal is transmitted to the salience system which in turn generates a control signal to engage brain areas mediating attentional, working memory, and action selection processes (while disengaging the default mode network). Critically, these switching mechanisms help focus attention on stimuli that signal deviant events or undesirable outcomes, as a result of which they take on added significance or saliency (Ullsperger et al., 2010).

Orienting to salient events or states that are associated with motivational significance could take various guises. One may orient attention to extraneous stimuli that call for action updating in order to secure valued outcomes and avoid undesired outcomes (stimuli that are novel, infrequent, deviant, unexpected, threatening, etcetera; or that serve as instructed targets or distracters); one may become receptive to induced emotions or affective states that call for approach or avoidance; or one may seek to monitor one’s internal and external milieu for signals that register as a risk for undesirable outcomes (e.g., slips of action, performance errors; response capture, action conflict; negative feedback, punishment, lack of expected reward). In general, the salience system appears to be central to monitoring for specifically those motivationally important changes that require autonomic regulation (Critchley, 2009).

The AIC and the ACC have direct anatomical connections to the autonomic nervous system, mostly via brainstem nuclei that provide feedback on bodily states and changes in autonomic arousal (Craig, 2002). In particular, these cortical areas have robust connectivity to the locus coeruleus/norepinephrine (LC/NE) system involved in boosting and maintaining phasic and tonic arousal (Aston-Jones and Cohen, 2005). The LC is the main NE-generating nucleus in the brainstem, and the LC/NE system is central to regulating the sympathetic discharge and the inhibition of parasympathetic tone in arousal responses. Indeed, salient events are consistently associated with increased pupil-dilation response and skin conductance and with decelerated heart-rate, the more so for more unexpected events such as errors (Critchley, 2005). Taken together, this new understanding of the AIC within the context of the salience system provides a starting point to study communalities in inter-individual differences in error awareness and in the ability to selectively attend to motivational relevant events, as discussed in the next sections.

### Errors as salience signals

Empirical (Notebaert et al., 2009) and theoretical work (Ullsperger et al., 2010) has emphasized notable parallels between the processing of errors and of other rare/deviant/novel stimuli (or otherwise potentially significant or motivationally relevant events). Erroneous outcomes and other performance problems can be seen as salient events because of their infrequent occurrence and their usefulness as learning signals. They trigger a reflex-like orienting response in the salience network, which is accompanied by a cascade of central and autonomic nervous system reactions associated with increased autonomic arousal as needed to recruit the mental and physical resources required for adaptive action. This reflex-like orienting signal in the salience networks may act as an internal monitoring signal, timely informing the organism of behavioral changes that need to be made.

Meta-analyses have shown that the AIC and ACC are consistently reported to be co-activated during errors and other instances when performance monitoring becomes necessary (Ridderinkhof et al., 2004; Klein et al., 2007). Consistent with these observations, indices of autonomic arousal co-vary with conflicts, errors, and feedback. For instance, error commission results in robust heart-rate deceleration and enhanced pupil-dilation responsivity, and these changes (that represent the recruitment of arousal so as to prepare the organism for adaptive action) tend to correlate with activity in the AIC and ACC.

### Error awareness versus error blindness

Error signals sometimes go unnoticed – they might need an appropriate potential in order for them to alert and engage the salience system and tip the balance between other related large-scale brain systems. For example, in order to be amplified into an orienting reaction in the salience network, error signals might need to surpass a certain energy threshold, or be accompanied by sufficient levels of physiological arousal. Performance errors are almost routinely registered in ACC, even if the individual does not consciously recognize the error as such (Nieuwenhuis et al., 2001; Endrass et al., 2007) but subsequent post-error slowing and changes in autonomic activity are observed only when subjects were aware of their error (Overbeek et al., 2005; Wessel et al., 2011). Error awareness has been found to engage specifically the right AIC but seems to place demands on bilateral anterior insula when applying a less conservative threshold (Klein et al., 2007). Specifically neurons situated in the anterior part of the insula are hypothesized to play a role in error awareness (Ullsperger et al., 2010). Activation of these anterior neurons is also observed during interoceptive awareness and the regulation of the body’s homeostasis (Critchley et al., 2005), whereas neurons in the posterior part of the insula are thought to be involved in somatosensory or proprioceptive perception (Craig, 2002).

### Error awareness vis-à-vis orienting to oddballs

Event-related potential (ERP) studies have highlighted two electrocortical components that can be observed when people make errors: the error(-related) negativity (*N*_E_ or ERN) and the error positivity (*P*_E_; Falkenstein et al., 1999). The *N*_E_ is believed to reflect activity in the dorsal ACC when the detection of a performance error signals the loss of anticipated reward and the need for adjustments to achieve action goals; the *P*_E_ appears to reflect the conscious recognition of the fact that an error was committed (for review, see Overbeek et al., 2005). A perspective on the functional significance of the *P*_E_ in terms of error salience or motivational significance suggests that the *P*_E_ reflects processes similar to those expressed in another ERP component, the classical P3b (Polich, 2007). The events that give rise to a P3b can vary widely (from salient, novel, or rare stimuli to the absence of expected stimuli) but appear to have in common that they are motivationally significant, that is, they should motivate the individual to initiate or change a course of action in order to keep performance at an optimal level (Ridderinkhof et al., 2009). According to recent views, the P3b comprises the electrocortical expression of the response of the LC/NE system to the preliminary outcome of internal decision making processes and the consequent effects of the noradrenergic potentiation of information processing (Aston-Jones and Cohen, 2005; Nieuwenhuis et al., 2005).

A robust finding is that P3 amplitude is inversely related to target probability in oddball tasks (requiring the detection of distinct infrequent target stimuli or oddballs which are embedded in a series of frequently presented non-target or standard stimuli; e.g. (Duncan-Johnson et al., 1984). Moreover, P3s to oddballs are more enhanced when the target stimulus is embedded in a train of non-target stimuli rather than in a train of other targets (Squires et al., 1976). Rather than being attributable to target probability *per se*, these P3 effects are crucially mediated by target-to-target interval (TTI) duration (Croft et al., 2003). The effect of TTI on P3b amplitude was observed to co-vary with the amplitude of the *P*_E_ (Ridderinkhof et al., 2009), supporting the notion that the *P*_E_ and P3b reflect similar neurocognitive processes possibly involved in the conscious processing of motivationally significant events. In an earlier combined neuroimaging ERP study, Horovitz et al. (2002) found similar parametric effects of TTI on P3 amplitude.

Several groups have examined brain regions critical for identifying and responding to oddball-targets (Horovitz et al., 2002; Liebenthal et al., 2003; Kiehl et al., 2005). Areas sensitive to the parametric effects of TTI were found in ACC and AIC (as well as parietal cortex and the thalamus), confirming the suggestion that regions implicated in generating the P3 (Soltani and Knight, 2000; Stevens et al., 2005) coincide with the observed activations in AIC in error awareness (Klein et al., 2007).

### Current aims

The studies reviewed above strongly suggest a role for the AIC in orienting to salient events, such as errors (when recognized as such) and relevant infrequent events (when occurring unexpectedly). The current study aims to test the involvement of the AIC in both processes directly. The notion that conscious detection of an error triggers an orienting response toward a motivationally significant event, similar to the orienting response to a rare target stimulus, would gain considerable support if it could be shown that the hemodynamic response during error awareness overlaps with the parametric effect of TTI during an oddball task. The orienting response toward the detection of a deviant target was examined using an oddball task, using a TTI manipulation known to parametrically affect specifically the processing of motivational salience (Nieuwenhuis et al., 2005). Thus, here the TTI manipulation was introduced into the oddball task to tap the process salience processing.

We aim to explore whether the AIC activation for error awareness is related to salience processing by testing for activation overlap in subjects undergoing two different task settings: in the same scanning session, the same subjects completed an antisaccade task with self-evaluation of each antisaccadic response; a task frequently used to study error awareness as it typically elicits a considerable number of performance errors, of which approximately 50% remain unaware (Nieuwenhuis et al., 2001). The advantage of acquisition of both the antisaccade and the oddball task in one scanning session is that brain activation on these two tasks can be compared not only at the group-level, but can also be tested within each participant’s brain activation. This yields a more precise comparison of the exact spatial distribution of the brain activation between the two cognitive processes. We predict that the hemodynamic response during aware (but not unaware) errors in the AIC overlaps with the oddball TTI effect. Specifically, we hypothesize that AIC of an individual, who engages to a higher degree in consciously detected errors also engages to a higher degree in the processing of deviant targets after a longer interval.

## Materials and Methods

### Participants

Fourteen healthy right-handed volunteers (12 females, mean age 21.2 ± 1.79)^1^ with normal or corrected-to-normal vision participated in the experiment after giving written informed consent according to the Helsinki Declaration. They were paid 50 Euros for participation. None of the participants had a history of neurological or psychiatric disorders or eye-problems nor was taking medications influencing the central nervous- or cardiovascular systems. Participants were administered two tasks (antisaccade and oddball in counterbalanced order) within one scanning session.

### Tasks

#### Oddball task

**Figure 1:**
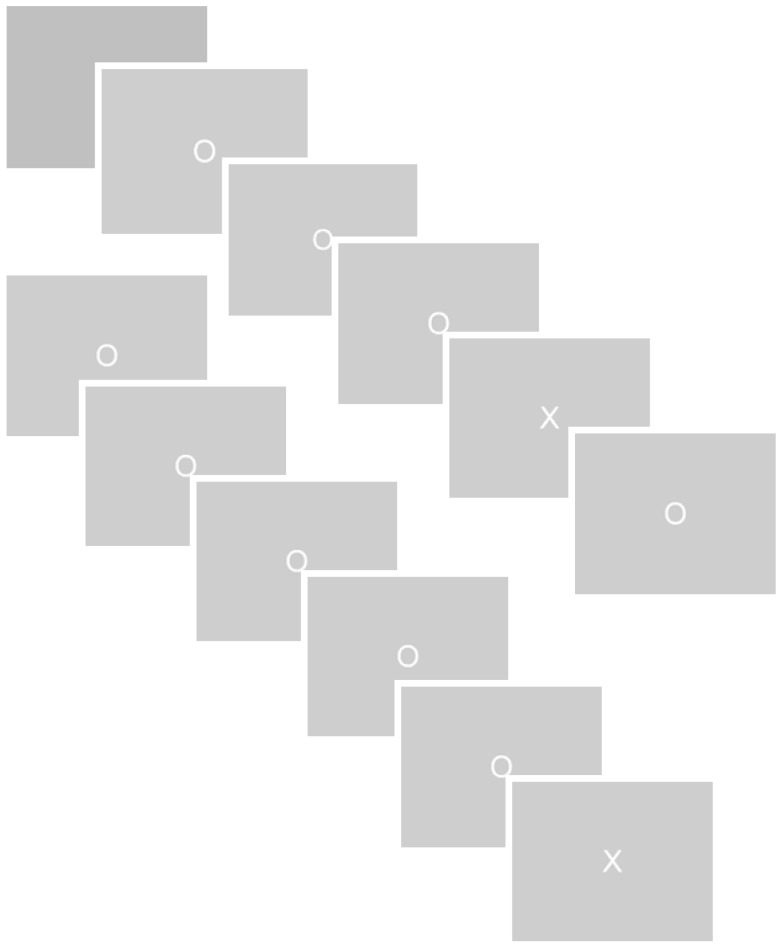
**Oddball task**. A series of non-target (0) and target (X) images was presented against a gray background. The target- and non-target ranged from 3 to 17 non-targets between targets. Participants were to react as quickly and accurately as possible to targets only by pressing a button with their index finger.

The orienting response was examined in an oddball task, using a TTI manipulation shown to parametrically affect salience processing specifically as reflected in the P3 (Ridderinkhof et al., 2009). The oddball task comprised a series of non-target and target stimuli that were presented for 100 ms on a computer screen in white uppercase letters (Os and Xs respectively, 2.5 cm × 2.5 cm = 1.16° × 1.16°) against a gray background. Between stimuli a white fixation cross appeared (0.30 cm × 0.30 cm, 0.14° × 0.14°) for 1400 ms. Three experimental blocks, each lasting 8.15 min, were presented to the subject, each of which contained 300 non-targets and 30 target stimuli. The sequence of target and non-target trials was varied in such a way that 15 TTI (the number of non-targets between two targets) were created. These TTIs ranged from 3 to 17 non-targets between targets. The sequence of these 15 TTI conditions within blocks was determined randomly by the computer. Participants were instructed to react as quickly and accurately as possible to targets only using a button of an fMRI-compatible response box with their index finger. No reaction was required to the presentation of non-targets. For the fMRI analysis of the effect of inter target interval on BOLD signal, the 15 TTIs were divided *post hoc* into three TTI conditions TTI-1, TTI-2, and TTI-3. TTI-1 comprised 3–7 non-targets between targets, TTI-2 comprised 8–12 non-targets, and TTI-3 comprised 13–17 non-targets. The temporal order of stimuli is depicted in Figure 1.

#### Antisaccade task

We examined unaware and aware errors in an antisaccade task with self-evaluation of each antisaccadic response, a task that typically elicits a considerable number of performance errors, of which approximately 50% remain unaware (Nieuwenhuis et al., 2001). Participants were instructed to fixate on a central target and generate an immediate eye movement away from an abrupt peripheral target to its mirror location on the opposite side of the screen without making an eye movement to the peripheral target itself.

A trial was classified as an error, when the participant looked at the peripheral target, even when this error was immediately corrected. To increase the error rate, a brief precue was presented at the position where the gaze should be directed to (Fischer and Weber, 1996; Klein et al., 2007). To reduce predictability, the precue was presented at the position of the following peripheral stimulus in 33% of the trials.

After the eye movement, participants were to indicate with a button-press whether their antisaccadic response was correct (immediate eye movement to the other side of the screen) or incorrect (initial eye movement toward the target). The erroneous responses participants had rated as incorrect were classified as aware errors and erroneous responses rated as correct were classified as unaware errors. If the erroneous eye movement was redirected to the correct (opposite) side of the screen, the response was labeled “corrected error.”

**Figure 2:**
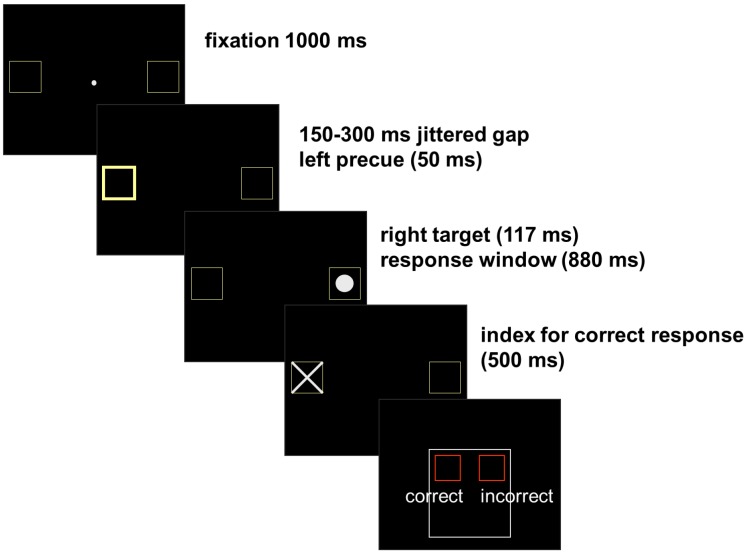
**Antisaccade task: participants were instructed to fixate on a central target and generate an immediate eye movement away from an abrupt peripheral target to its mirror location on the opposite side of the screen without making an eye movement to the peripheral target itself**. After the response a cross appeared in the correct square indicating the correct gaze direction. Participants were to evaluate their performance by pressing one of two buttons of an fMRI-compatible response box. An initial eye movement toward the peripheral target was classified as an error. The erroneous responses participants had rated as incorrect were classified as aware errors and erroneous responses rated as correct were classified as unaware errors.

Participants completed 3 blocks of 100 antisaccade-trials, each lasting 11 min. For assessment of the pupil response, light flux was calibrated to equal luminance across trials with the program Colorfacts 7 and the color calibration system “EyeOneMonitor^2^” and tested for equal pupil luminance response across precue conditions. There was no significant difference in pupil-dilation between trials with (0.4 ± 1.1) and without precue [0.4 ± 1.2; *t*(22) = 0.01, *p* < 0.995]. Light in the scanning environment was constrained to video presentation of stimuli against a black background. The trial started with a central fixation dot surrounded by two square outlines (each subtending 3.8° visual angle; distance from fixation 12.4°; display-duration 1000 ms). After a 150–300-ms jittered fixation gap, the peripheral target (a white circle subtending 2.9°) was unpredictably presented for 117 ms in the left or the right square. To induce erroneous responses a precue was presented in 50% of the trials, briefly (50 ms) thickening the outlines of the square at the opposite side of the target and validly indicating the target location. After a response window (of 880 ms) a cross appeared (for 500 ms) in the correct square indicating the correct gaze direction. Participants were to evaluate their performance (within 1500 ms) by pressing one of two buttons of an fMRI-compatible response box. On trial number 20, 40, 60, and 80, an instruction screen (duration: 2 s) appeared, reminding participants to keep saccading at fast pace. A black screen with jittered duration (16, 500, 1000, 1500 ms) was displayed between trials and 10% of the trials were “null events” (fixation-only trials of 5952 ms). The temporal order of stimulus presentation is displayed in Figure 2.

### Behavioral data acquisition and analysis

#### Oddball task

Stimuli were presented on a 66 cm × 88 cm screen, placed at a 4-m viewing distance at the front end of the scanner and seen through a mirror above the participants’ heads. Stimuli were presented and button-press responses (from an MRI compatible response box) were recorded with a presentation PC (Neurobehavioral Systems^3^, Albany, NY, USA), that was connected to the MRI-scanner allowing for the time locking of stimuli, responses, and fMRI image acquisition. Two generalized linear model repeated-measures analyses of variance (ANOVAs) were used to investigate accuracy and reaction time in the oddball task. The independent variable for this analysis was TTI. TTIs were collapsed together into three TTI bins (3–7, 8–12, and 13–17 consecutive non-targets).

#### Antisaccade task

Oculomotor, pupil, and button-press responses were recorded with two interconnected PCs: an eye-tracker PC (ViewPoint EyeTracker, Arrington Research)^4^ and a presentation PC (Neurobehavioral Systems, see text footnote 3, Albany, NY, USA). Both PCs were connected to the MRI-scanner allowing for the time locking of stimuli, responses, and fMRI image acquisition. The participant’s left eye was continuously monitored with an MRI compatible infrared oculographic limbus tracker (Resonance Technology, Inc.)^5^ attached to the head coil and placed 3 cm beneath the participant’s left eye. The eye-tracker registered eye movements, aspect ratio, and diameter of the pupil with a sampling rate of 60 Hz along with scanner pulses and stimulus onsets. Before the scan, a nine-point calibration was performed and calibrated eye position was slip corrected during the task to eliminate slow drifts. Calibration and stimuli were presented on a 66 cm × 88 cm screen, placed at a 4-m viewing distance at the front end of the scanner and seen through a mirror above the participants’ heads. Saccade onsets, amplitudes, and directions were detected with in-house Java-based software^6^ using minimum amplitude (>1.5°) and velocity (>30°/s) criteria and were subsequently double-checked by two raters. In line with common definitions (Fischer et al., 1993) we excluded trials in which subjects initiated saccades faster than 80 ms after target appearance [3.3 ± 4.1% (SD) of all trials], trials in which subjects were looking away from fixation during target presentation (2.7 ± 3.9%), blinked during target appearance (0.6 ± 1.2%) and trials for which the eye movement data were not interpretable due to poor quality of the eye-tracker signal (5.0 ± 4.3%).

### fMRI acquisition and analysis

#### Acquisition

Functional images during the oddball- and the antisaccade task were acquired in the same subjects in the same scan session on a Philips (Philips, the Netherlands) 3 T MRI system equipped with echo planar imaging (EPI) capabilities using a standard head coil for radio frequency transmission and signal reception. Functional scans of the entire brain were acquired with a single-shot, gradient-recalled EPI sequence parallel to the AC–PC plane (TE/TR = 28/2000 ms; 30 axial slices; slice thickness 3 mm; interslice gap 0.3 mm; voxel size 3 mm × 3 mm × 3 mm; FOV = 222 mm × 2 mm; 96 × 96 in-plane resolution/matrix size, 90° flip angle). The first two volumes were discarded to allow for T1 equilibration effects. The duration of the oddball task was three times 8.15 min (245 scans per scanblok), the antisaccade task was three times 11 min (335 scans per scanblok). High-resolution anatomical images were subsequently acquired using a 3-D T1-weighted scan in steady state sequence (TE/TR = 4.6/9.69 ms; 182 sagittal slices; slice thickness 1.2, interslice gap 0.3 mm; voxel size 1 mm × 1 mm × 1 mm cubic; FOV = 25 cm × 2 cm; 256 × 2 in-plane resolution, 8° flip angle, sagittal orientation).

#### Preprocessing and GLM

Preprocessing of the functional data and calculation of the contrast images for statistical analysis was done with FEAT (FMRI Expert Analysis Tool) Version 5.63, a part of FSL (FMRIB’s Software Library)^7^. Functional images were realigned to compensate for small head movements, slice-time corrected, spatially smoothed with a 5-mm full-width half-maximum Gaussian kernel, filtered in the temporal domain using a high-pass filter with a cutoff frequency of 1/50 Hz to correct for baseline drifts in the signal and prewhitened (Woolrich et al., 2009). For each experimental run of each participant, the overall activity was modeled as evoked by the targets (which were associated with one of three TTI conditions: TTI-1, TTI-2, and TTI-3; see task description), and by the correct responses and error commissions in the antisaccade task (two levels: aware errors versus unaware errors). The three levels (TTI-1, TTI-2, and TTI-3) in the oddball task were statistically compared first by fitting a linear model describing a linear signal increase from TTI-1 to TTI-2 to TTI-3), and second by subtracting TTI-1 from TTI-2, TTI-1 from TT-3, and TTI-2 from TTI-3. Each regressor in the oddball task and in the antisaccade task was convolved by a prototypical synthetic hemodynamic response function and its first derivative. To remove any artifactual signal changes due to head motion, six parameters describing the head movements (three translations, three rotations) were included as confounds in the model. In the second-stage analysis participants were treated as a fixed factor to concatenate the three experimental runs. Contrasts pertaining to the main effects constituted the data for the third-stage (mixed effect) analysis, where the significance of observations was determined across the group of 14 subjects using FLAME 1 and 2 (FMRIB’s Local Analysis of Mixed Effects; Smith et al., 2004). For each whole-brain comparison of the target interval conditions in the oddball task we computed the initial statistical test with FSL-FEAT (FMRIB’s Software Library; see text footnote 7), and thresholded the resulting *z* statistic image to show which voxels or clusters of voxels are activated at a particular significance level. We selected cluster thresholding, and used a *z* statistic threshold to define contiguous clusters. Each cluster’s estimated significance level, corrected for whole-brain multiple comparisons using Gaussian random field theory (GRFT), and was compared with the cluster probability threshold. Significant clusters were then used to mask the original *z* statistic image for later production of color blobs. A cluster of voxels was considered significantly active if it passed the threshold of *z* = 2.3 and *p* = 0.0.05. This method of thresholding is an alternative to voxel-based correction, and is normally more sensitive to activation.

#### Comparative analyses

Participants had completed both the antisaccade and the oddball task within one scanning session. The advantage of this acquisition is that brain activation on these two tasks can be compared not only at the group-level, but can also be tested within each participant’s brain activation, i.e., in his native functional space. For a given participant this native functional space is an image with brain activation acquired on that particular subject. The image is not yet transformed into a standard reference image, as for example the MNI brain from the Montreal Neurological Institute that defined a standard brain by using a large series of MRI scans on normal controls, representative of the population. This yields a more precise comparison of brain activation between the two cognitive processes. Four types of comparison were applied: spatial overlap analysis at the group-level, contrast masking analysis, Region of interest (ROI)-based correlation analysis and ROI-based ANOVA-analyses of average regression weights across tasks, within subjects.

#### Step 1: group-level spatial overlap analysis

**Figure 3:**
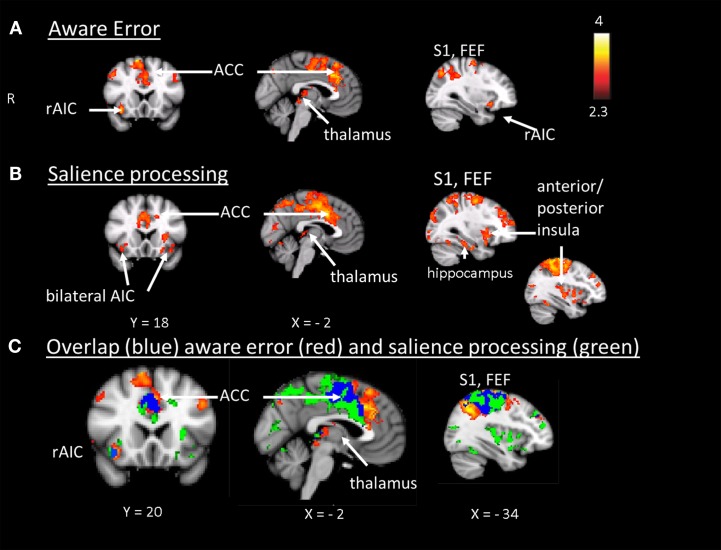
**(A)** Aware error: statistical parametrical map of difference in BOLD activation between aware and unaware errors. Red and yellow voxels represent clusters of significant BOLD signal increase. **(B)** Salience processing: statistical parametrical map of difference in BOLD activation for the parametrical oddball. Red and yellow voxels represent clusters of significant BOLD signal increase. Renderings (on MNI stereotactic space) are thresholded at *z* = 2.3 and *p* = 0.05. **(C)** Overlap: plotted overlap between BOLD activation in the same subjects and the same scan session during aware errors and during the salience processing Note: R, right; ACC, anterior cingulate cortex; AIC, anterior insula cortex; FEF, frontal eyefields; S1, somatosensory cortex.

In step 1 we plotted mean group activation during aware (compared to unaware) errors in the antisaccade task on top of the mean group activation that was elicited by oddballs and sensitive to parametric TTI effects in the oddball task. This yielded a map illustrating the spatial localization of brain areas showing increased amplitude of the hemodynamic response to aware errors and to target stimuli with a parametrically increasing TTI (Figure 3-Overlap).

#### Step 2: contrast masking analysis on single subject level

In step 2 we tested within each participant at the whole-brain level for overlapping clusters of activation between the aware versus unaware contrast and for oddball-target detection (which was associated with parametric TTI effects). To this purpose we applied FMRIB’s Local Analysis of contrast masking (Smith et al., 2004).

With the FSL-function of contrast masking, one can set up the masking of contrasts by other contrasts. After thresholding of all contrasts has taken place one can further threshold a given *z* statistic image by masking it with non-zeroed voxels from other contrasts. Non-zeroed voxels are voxels with have passed the cluster threshold of *z* = 2.3 and *p* = 0.05 in the contrast. This means that of the voxel clusters, which passed thresholding in the first contrast of interest, only those, which also survived thresholding in the other contrasts, are kept. Aim of this analysis is to detect overlapping clusters of voxels that survive within one participant both the threshold for the awareness contrast and the threshold for oddball-target detection (which was associated with parametric TTI effects).

First the initial statistical test was carried out for the error awareness task. The resulting *z* statistic images were thresholded to show which contiguous clusters of voxels were activated in each participant at the statistic threshold of *z* = 2.3 and *p* = 0.05 in the contrast aware versus unaware error (Smith et al., 2007). The result was a thresholded *z* statistic image for aware as compared to unaware errors, that constituted all contiguous clusters of voxels that had survived the cluster threshold of *z* = 2.3. In the next step the contrast for the oddball-target detection which was associated with activation after the longest target tot target interval) was computed at statistic threshold of *z* = 2.3 and *p* = 0.05, within the “mask” of the error awareness contrast. This means that of the oddball-target clusters which passed *z*-thresholding, only those which also survived *z*-thresholding in the aware versus unaware contrast are kept.

Thus, we constrained our search to activation in the aware versus unaware contrast which was also sensitive to oddball-targets which were associated with the longest TTI.

**Figure 4:**
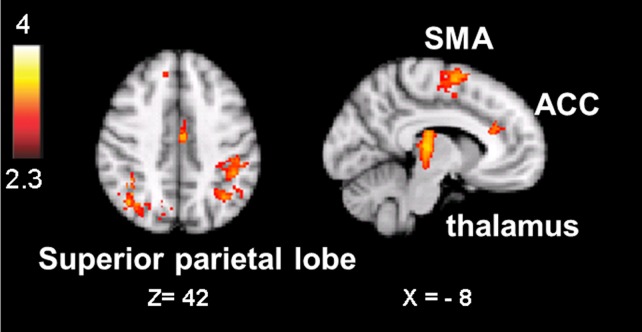
**Spatial overlap map of clusters of activation on group-level that survived, within each participant’s native functional space, both the threshold for the awareness contrast and the threshold for salience processing**. Analyses were we constrained by creating for each individual an “error awareness brain mask” within which activation was sensitive to salience processing. Renderings (on MNI stereotactic space) are thresholded at *z* = 2.3 and *p* = 0.05. Note: ACC, anterior cingulate cortex; SMA, supplementary motor area.

The result is a conservative analysis: brain structures with few or distributed active voxels will not survive thresholding. The resulting spatial overlap maps of each subject were subsequently fed into a group-level analysis. For this mixed effect analysis, FLAME 1 and 2 (FMRIB’s Local Analysis of Mixed Effects; Smith et al., 2004) was used, in which the significance of activation common to error awareness and oddball-target detection associated with parametric TTI effects was computed across the group of all 14 subjects. We report a cluster-corrected threshold of *p* < 0.05 corrected for whole-brain multiple comparisons (using GRFT). The result is a precise spatial map depicting “error awareness areas” that are also sensitive to oddball-target detection associated with parametric TTI effects (see Figure 4).

#### Step 3: ROI-based correlation analysis

The AIC has been found associated to error awareness more consistently than the ACC. Hence, the AIC constituted an *a priori* ROI. Specifically, we were interested in determining whether those individuals who engaged the AIC to a greater extent during consciously detected errors also engaged this area more strongly when processing deviant targets after longer TTI’s. Therefore, in step 3 we extracted the hemodynamic response of each participant’s AIC to error awareness (aware error-unaware error) and to parametric TTI effects during target detection (linearly increasing parametric hemodynamic response across TTI, TTI-2, and TT-3), and correlated these two extracted difference scores across participants. As error awareness has previously been shown to engage only the anterior part of the insula and may furthermore engage the left and right AIC differentially (Klein et al., 2007). ROIs were defined for the anterior and posterior insula and for the left and right hemisphere separately. To test for the specificity of the AIC in orienting to salience, we contrasted the AIC to posterior insula cortex (PIC) activation.

**Figure 5:**
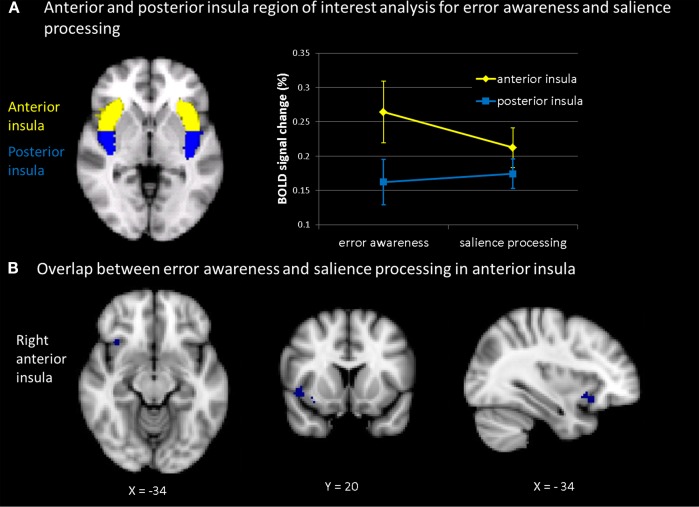
**(A)** Mean percent BOLD signal change within subjects across tasks (for the contrast aware errors as compared to unaware errors; and for the contrast of salience processing, i.e., linear signal increase across inter-target interval) in anterior insula and posterior insula (thresholded at *z* = 2.3 and *p* = 0.05). Participants showed during both error awareness and salience processing a significantly higher percent signal change in the anterior than in the posterior insula. The main effect of task indicated no differences in percent signal change in the insula between error awareness and the processing of motivationally significant events **(B)**: plotted overlap in the anterior insula within subjects across tasks (for the contrast aware errors as compared to unaware errors; and for the contrast of salience processing, i.e., linear signal increase across inter-target interval).

Definition of ROIs was based on the MNI structural atlas of the FSL-atlas toolbox and available literature on neurosurgical landmarks (Mazziotta et al., 1995; Ture et al., 1999; Eickhoff et al., 2007). For anterior and posterior masks, coordinates were taken from Brooks et al. (2005). The vertical border between anterior and posterior portions of the insula was chosen such that the AIC seed subtended the three principal short insular gyri (anterior, middle, posterior) and the accessory and transverse insular gyri, all anterior to the insular sulcus (border for right and left insular cortex at *y* = 1.3, see Figure 5A). Percent signal change in bilateral AIC seeds was extracted for each subject for the aware versus unaware error contrast from the antisaccade task and for the TTI-3 minus TTI-1 contrast from the oddball task. Since we were interested specifically in whether the insula was engaged, within participants, in error awareness as well as in oddball processing, we computed bivariate correlations between percent signal change in AIC (and PIC) during aware errors and during interval-related target detection across participants. The predicted correlations were tested one-sided. Pearson correlation coefficients and *p*-values are presented. The resulting correlation maps show the relation between signal changes derived from bilateral AIC (and PIC) seeds as induced by aware (compared to unaware) errors and by target detection at long (compared to shorter) TTI’s.

Step 4: ROI-based ANOVA-analyses of average regression weights (across tasks, within subjects, for each voxel). Within subjects and across tasks (the error awareness task and the oddball task) we performed analysis of regression weights using FSL’s Featquery signal change processing tool (Analysis group, FMRIB, Oxford, UK)^8^. Featquery was conducted to interrogate signal change of *a priori* ROIs, previously defined by the literature reviewed, the anterior and posterior parts of insula cortex. After transforming the anterior and posterior insula masks into the native low resolution space, Featquery extracted regression weights (parameter estimates) and converted them to percent signal change values. This is achieved by scaling the *P*_E_ values by (100^*^) the peak-peak height of the regressor and then by dividing by the mean image from fil filtered_func_data. This analysis yielded mean statistical values of signal change across the time series with the anterior and posterior insula. In the next step we fed these values into group-level ANOVA analysis (SPSS)^9^ to compare activation in anterior insula and posterior insula within subjects across tasks.

## Results

### Behavior

#### Antisaccade task

Mean error rate was 27.5 (SD: 15.5%). Erroneous responses were initiated faster than correct responses [190 versus 282 ms; *t*(13) = 7.1; *p* < 0.001]. Participants were aware of roughly half of their errors; the other half went unnoticed [13.6 versus 13.9%; *t*(13) = −0.063; *p* = 0.95]. In 74.8% of errors, participants immediately corrected their erroneous response with an eye movement to the correct location. Unaware and aware errors were similar in mean latency [186 versus 194 ms; *t*(13) = 0.40; *p* = 0.69]. Yet, unaware errors were corrected significantly more often than aware errors [93.1 versus 63.5%; *t*(13) = 2.8; *p* < 0.013]. False alarm rates below 5.0% indicated that participants rarely reported an error when they had made a correct antisaccade.

#### Oddball task

For the oddball task, mean reaction time for correct target detection responses was 317 ms. RT did not vary as a function of TTI, *F*(2,26) = 1.49, *p* = 0.25. Overall accuracy of target detection was 98.7% and did not vary systematically as a function of TTI, *F*(2,26) = 0.034, *p* = 0.97.

### fMRI activation patterns

#### The antisaccade task: aware versus unaware errors

**Figure A1:**
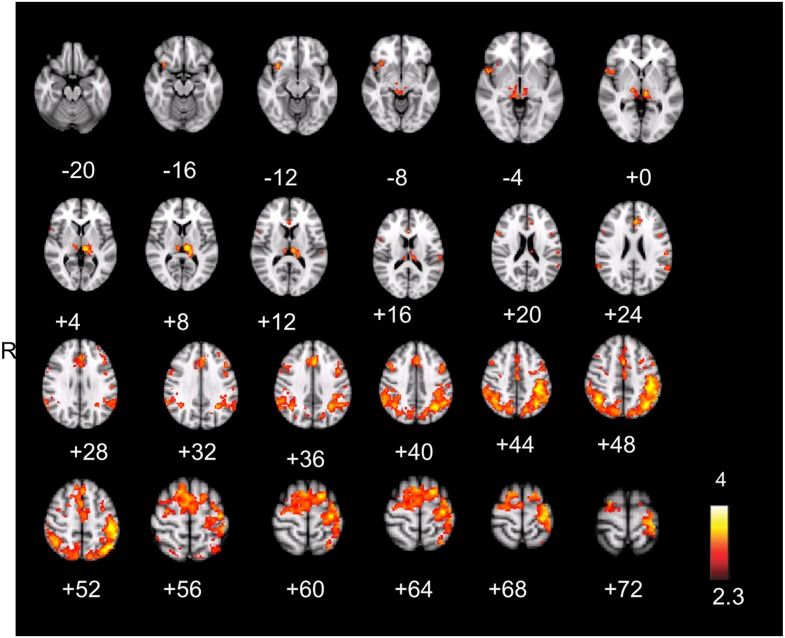
**Neural activation on aware errors**. Statistical parametrical map of difference in BOLD activation between aware and unaware errors. Red and yellow voxels represent clusters of significant BOLD signal increase across all subjects. For a full list of activated regions (*z* > 2.3, whole-brain cluster-corrected, *p* < 0.05), see Table A1.

Compared to unaware errors, aware errors yielded significantly increased activation in right AIC, dorsal ACC, bilateral pre- and postcentral gyrus (somatosensory cortex), bilateral frontal eyefields, superior parietal lobules, and bilateral thalamus (see Figure 3A; also Figure A1; Table A1 in Appendix).

#### The oddball task and salience processing: interval effects on target detection

**Figure A2:**
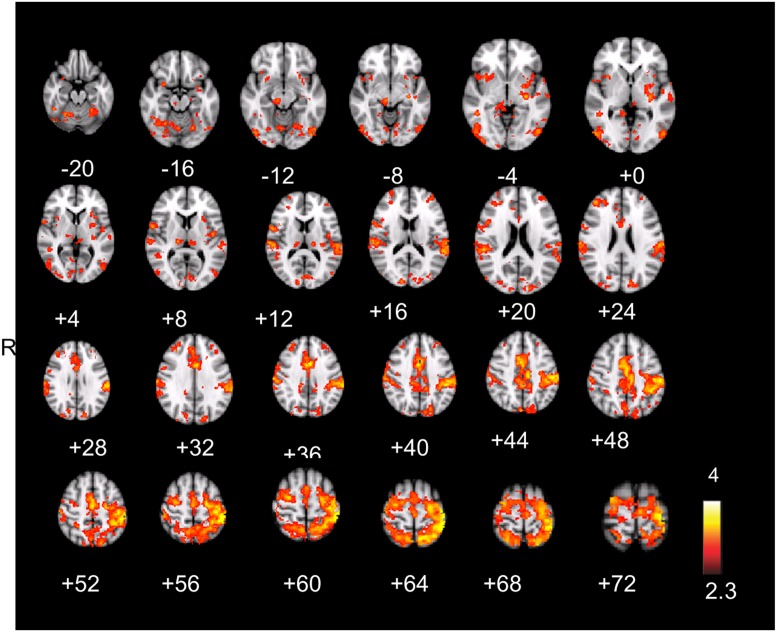
**Illustration of brain areas showing increasing amplitude of the hemodynamic response to target stimuli with longer target interval**. Target interval effects were found in numerous brain structures, including bilateral thalamus, bilateral anterior insula, dorsal anterior cingulate, supplementary motor area, dorsolateral prefrontal cortex, bilateral middle temporal gyri, bilateral pre- and postcentral gyri (somatosensory cortex), bilateral inferior and superior parietal lobules, parietal occipital junction, superior/middle and inferior frontal gyrus, precuneus, and bilateral cerebellum. The legend shows *z*-score value associated with the color map. The statistical parametric map has a threshold of *z* > 2.6; *p* < 0.05 (cluster-corrected). For a full list of activated regions, see Table A2.

**Figure A3:**
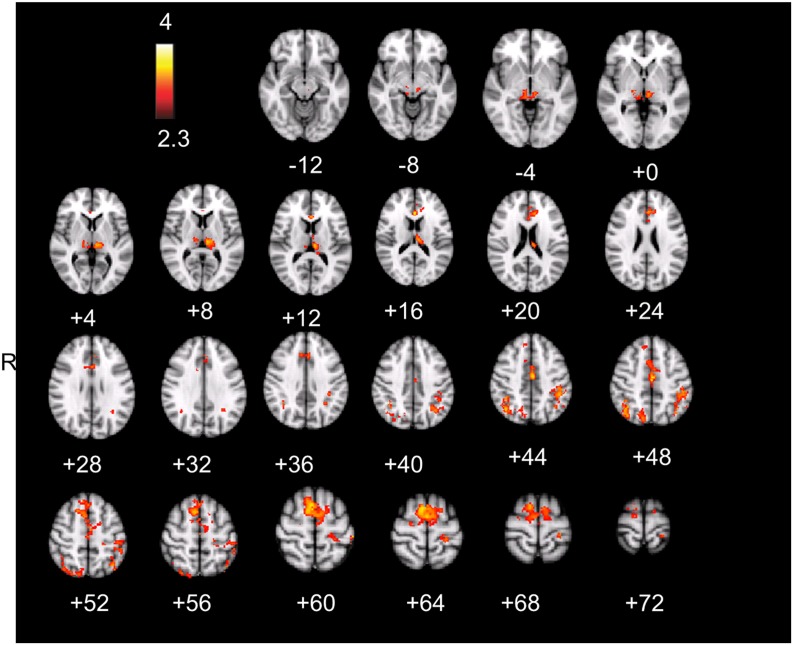
**Statistical parametrical map of hemodynamic response varying in each individual with aware errors and with the interval effect on target detection**. Red and yellow voxels represent clusters of significant BOLD signal which passed the thresholding in the target interval contrast (ITI3–ITI1) and also survived thresholding in the awareness contrast (aware versus unaware errors). Four major brain areas were involved in both contrasts: bilateral thalamus, supplementary motor area, rostral cingulate, and in bilateral parietal lobule. Furthermore, overlapping activations were found in the precuneus and lateral occipital gyrus.

The parametric effect of interval length (TTI) on the detection of an oddball-target was observed in a number of areas, including AIC and PIC, dorsal ACC, supplementary motor area, pre- and postcentral gyri (somatosensory cortex), inferior and superior parietal lobules, and the thalamus, mostly bilateral (see Figure 3B; also Figure A2; Table A2 in Appendix). TTI-3 minus TTI-1 contrast analysis (subtracting the shortest interval length from the longest interval length) yielded highly similar activation patterns (see Figure A3 in Appendix). These regions have been observed previously to be active not only during target detection but also as a parametric effect of target interval (Kiehl and Liddle, 2003; Kiehl et al., 2005; Stevens et al., 2005).

#### Group-level spatial overlap analysis

The hemodynamic response during aware errors (compared to unaware) errors showed commonalities and differences with salience processing (see Figure 3-Overlap). Overlapping activation was observed in the right AIC, dorsal ACC, somatosensory cortex and precentral gyrus (frontal eyefields), thalamus, and brainstem. Compared to error awareness, salience processing additionally yielded increased activation in the left AIC, bilateral PIC, hippocampus, and inferior and superior parietal lobules.

#### Contrast masking analysis

Active voxels which passed, in each individual participant, cluster-corrected thresholding both for the aware-unaware contrast and for salience processing were found in the supplementary motor area, dorsal ACC, inferior and superior parietal lobule (supramarginal gyrus, postcentral gyrus), and thalamus, mostly bilateral (see Figure 4; Table A3 in Appendix), as well as in the precuneus and lateral occipital gyrus (not shown). Notably, this analysis did not reveal overlapping voxels of activation in the AIC.

#### ROI-based correlation analysis

Mean percent signal change in the right and left anterior and posterior insula was extracted for all individual participants during aware (compared to unaware) errors. During error awareness, engagement of the left and right anterior part of the insula were strongly correlated (*r* = 0.82; *p* = 0.0001). Moreover, as depicted in Figure 5, participants who showed stronger engagement of the right and left anterior part of insula cortex during error awareness also showed stronger engagement of the right and left anterior part of insula cortex during target detection after longer compared to shorter TTI’s (*r* = 0.50; *p* = 0.03, one-sided). This association was observed in both the right (*r* = 0.44; *p* = 0.05, one-sided) and the left AIC (*r* = 0.51; *p* = 0.03, one-sided). Thus, participants who activated the AIC to a greater extent to aware compared to unaware errors also activated the anterior part of the insula to a greater extent to motivational salience (target stimuli after a longer compared to shorter sequence of non-target stimuli). Bilateral anterior and posterior insula activation during error awareness failed to correlate with oddball processing (target compared to standard stimuli) *per se* (right AIC: *r* = 0.29, *p* = 0.16, left AIC: *r* = 0.33, *p* = 0.13, right PIC: *r* = 0.10, *p* = 0.37, left PIC: *r* = 0.27, *p* = 0.18). These correlation coefficients for error awareness and motivational salience in the anterior insula were larger than the correlation coefficients in the anterior insula for error awareness and oddball processing *per se*. However, using the Fisher *r*-to-*z* transformation and the Meng test of two correlations with one variable in common from the same sample (Meng et al., 1992), the difference between these correlation coefficients of motivational salience and oddball processing did not reach significance in both tests (Fisher: *z* = 0.41, *p* = 0.34; Meng: *z* = 0.038, *p* = 0.485 for right AIC; *z* = 0.52, *p* = 0.3, Meng: *z* = 0.043, *p* = 0.483 for left AIC). Furthermore, the observed association was only observed in the anterior part of the insula; activation in the posterior part of the right and left insula showed no significant association between error awareness and TTI effects (bilateral PIC: *r* = 0.18; right PIC: *r* = −0.04; left PIC: *r* = 0.43). The difference between the correlation coefficients of error awareness and motivational salience in anterior insula (*r* = 0.5) and in posterior insula (*r* = 0.18) however failed to reach significance in both the Fisher and the Meng test (Fisher: *z* = 0.92, *p* = 0.18 (one tailed); Meng: *z* = 0.08, *p* = 0.468). In conclusion, we observe a tendency toward higher correlations between the two processes error awareness and motivational salience in the anterior insula than in the posterior insula and a tendency toward higher correlation between motivational salience and error awareness, than between oddball processing and error awareness, but the difference between the correlation coefficients does not reach significance level.

#### ROI-based ANOVA-analyses of average regression weights across tasks, within subjects

As can be seen in Figure 5A, a main effect of insula indicated differences in percent signal change between anterior and posterior insula [*F*(1,13) = 38.717, *p* < 0.0001]. The percent signal change values were analyzed using a mixed 2 × 2 ANOVA design with two within subjects variables (insula with two levels anterior and posterior; task with two levels error awareness and motivational significance). Participants showed during both error awareness and the processing of motivational significance a significantly higher percent signal change in the anterior insula than in the posterior insula. The main effect of task indicated no differences in percent signal change in the insula between error awareness and the processing of motivationally significant events [*F*(1,13) = 0.777, *p* < 0.394]. The test for interaction indicated that error awareness is associated with a marginally higher percent signal change in the anterior insula and a lower percent signal change in the posterior insula than the processing of motivational significance in the oddball task, trending toward significance [*F*(1,13) = 23.989, *p* < 0.067].

## Discussion

We report that error awareness shares anterior insula and cortico-thalamic circuits with target detection as modulated by TTI in a visual oddball task (referred to as “salience processing” in the remainder of the text).

Error awareness and salience processing showed activation overlap in six major brain areas: anterior insula, anterior cingulate, supplementary motor area, thalamus, brainstem, and parietal lobe. The findings of individual differences analysis of the *a priori* ROI AIC revealed that participants who activated the AIC to a higher degree to error awareness, also activated the AIC to a higher degree to salience processing. Within the AIC, interesting topographic differences were visible: error awareness activated predominantly the ventral AIC, whereas salience processing seemed to activate the AIC to a larger extent with maxima in the dorsal AIC, and with activation extending to PIC. The fine-grained contrast masking analysis within each participant’s brain activation confirmed this observation: within AIC non-identical neural ensembles seem to be robustly activated within the same subjects during error awareness and salience processing. Robust direct spatial overlap was visible in the dorsal ACC, the supplementary motor area, the thalamus, and the parietal lobes. The results of the ROI-based ANOVA-analyses of average regression weights show that within subjects the insula shows significant percent signal change in both error awareness and salience processing (no significant main effect of task), and that the anterior part of the insula is significantly more involved in both processes than the posterior part (significant main effect of insular sub regions). Furthermore there is a tendency toward more AIC involvement and less PIC involvement in error awareness than in salience processing in the oddball task.

Together, these outcomes suggest a similar functional phenomenon in the two different task settings. In particular, they show a shared functional insula-cortico-thalamic anatomy for error awareness and salience processing, with some subregional anterior posterior specialization within the insula, and ventral dorsal specialization within the anterior insula.

The advantage of the current approach lies in the acquisition of both the “error awareness antisaccade task” and the oddball task in one scanning session in the same subjects. Overlap in brain activation on these two tasks can be compared not only at the group-level, but can also be tested within the brain activation of each participant. This yields a more precise comparison of the exact spatial distribution of the brain activation between the two cognitive processes.

One potential disadvantage of this approach lies in high stringent thresholds applied to extract only voxel clusters that are robustly involved in both tasks in each participant’s brain activation. This threshold was chosen to account for noise in the individual data, but may lead to false negative results in small brain structures with activation in small voxel clusters.

Therefore, in order to gain a comprehensive picture of the overlap, three comparisons have been computed, and will be discussed below: (1) A whole-brain comparison with the plotted overlap of group-level activation patterns (for aware errors as compared to unaware errors plotted on parametrical effects of long as opposed to short ITIs in the oddball task); (2) a comparison showing the whole-brain group-level result of spatial overlap calculations (contrast masking of parametric target detection with activation clusters of error awareness) within each participant’s brain activation; (3) ROI analyses focused on AIC, the *a priori* structure of interest in error awareness. In the following sections we will discuss first the findings on the whole-brain level, and second the findings that focus on the AIC. The AIC findings are placed in the context of current views on its role within larger scale functional and structural brain networks.

### Widely distributed overlap

Error awareness and salience processing showed overlap in six major brain areas. In general, this widespread overlap suggests that both error awareness and salience processing seem to engage multiple, spatially distributed processing systems. The most parsimonious cognitive interpretation of the widespread overlap is that it reflects a greater capture or orienting of attention (for aware errors as compared to unaware errors, as well as for long as opposed to short TTIs). The widespread overlap is reminiscent of similar patterns reported by Kiehl and co-workers in relation to reflexive or automatic orienting processes, that have been shown to reliably activate an extensive neural network (Kiehl and Liddle, 2003; Kiehl et al., 2005; Stevens et al., 2005).

Halgren et al. (1980, 1995a,b) have argued that a widely distributed response to salient events may be “adaptive” in an evolutionary sense. Activating many potentially useful areas, despite the low probability that these regions are all immediately functionally necessary, may lead to superior incidental learning and performance. The results of the “contrast masking comparison” of error awareness and salience processing, with stringent thresholds, included regions believed to mediate attentional control, particularly for salient stimuli, including thalamus, ACC, supplementary motor area, and superior parietal lobule (Corbetta and Shulman, 2002). As the ACC is known to increase in activation during conditions involving conflict monitoring (Ridderinkhof et al., 2004), the current activation overlap may reflect a cumulative process of increasing target expectancy and error monitoring (Squires et al., 1976; Kiehl et al., 2000). Additionally, a number of studies suggest a role of the dorsal ACC and the thalamus in the generation of peripheral arousal (Critchley, 2005). Single cell recordings show that the thalamus seems to be the first to react to an oddball stimulus to elicit an arousal response via its connections with the dorsal ACC (Klostermann et al., 2006). The robust overlap in dorsal ACC and the thalamus in the current data-sets may reflect a central correlate of peripheral system reactions associated with the increased autonomic arousal as needed to recruit the mental and physical resources required for adaptive action to the detection of errors and rare targets. Taken these two lines of thoughts together, the overlapping signal in the dorsal anterior cingulate and thalamus may act as an reflex-like orienting and monitoring signal, timely informing, and preparing the central and the peripheral neural system for behavioral changes that need to be made.

In line with this interpretation, a number of studies suggested that anterior cingulate activation might influence norepinephrine modulation of P3 in oddball tasks (Nieuwenhuis et al., 2005). Nieuwenhuis and colleagues proposed that phasic norepinephrine activity as mediated by the brainstem nucleus Locus Coeruleus may serve to enhance future top-down mediated selective attention for salient stimuli. Nieuwenhuis et al. (2005) predict that TTI would enhance neural response in brain areas active during task-relevant target processing, whereas no modulation would be seen to motivationally insignificant stimuli, which is the pattern of results found in this study in both the detection of motivationally significant rare targets as well as the conscious detection of errors. This is in line with the striking parallels in the association of the *P*_E_ with error awareness and the association of the P3b with salience processing (Ridderinkhof et al., 2009). Notably, both the P3b and the *P*_E_ have been proposed to be related to phasic activity of the Locus Coeruleus/norepinephrine system as has the orienting response (Overbeek et al., 2005).

The imminent question whether the observed robust hemodynamic overlap within the participants’ native functional space in the thalamus, the parietal lobes, the dorsal ACC, and the supplementary motor area in the current study reflects activation in brain regions implicated in both generating the P3b (Soltani and Knight, 2000; Stevens et al., 2005) and the *P*_E_ (Klein et al., 2007) remains unclear. With the present fMRI study, it is not possible to definitely state whether the hemodynamics are related to one or another ERP “component.” Even with the currently observed overlap, it remains possible that the neural source of the P3 elicited by the parametric oddball task may differ from the neural source of the *P*_E_ elicited by error awareness. The degree to which this overlap in hemodynamic change reflects common neural sources of the P3 and the *P*_E_ is not certain and might be effectively addressed by future studies combining fMRI and ERP technologies. Another suggestion for future studies could be to experimentally manipulate both the awareness of the error and the motivational significance of the event within one task. If error awareness would trigger insula activation also in the absence of motivational salience, it would make sense to describe these as different processes with a shared functional anatomy. As such manipulations are not be workable in the classical error awareness antisaccade task, used by this and previous studies on error awareness (Nieuwenhuis et al., 2001; Endrass et al., 2007; Klein et al., 2007), one may transfer the question to perceptual awareness tasks (such as used by van Gaal et al., 2010) or to tasks investigating the reward prediction error (Schoenbaum et al., 1998; Schultz, 1998).

In the error awareness task we observed a higher proportion of behavioral adjustments of the antisaccadic response (i.e., reversing an initial prosaccade into a timely antisaccade) after unaware errors than after aware errors. In speculation, this could have an impact on the BOLD-contrast aware versus unaware errors. Behavioral corrections after unaware errors may theoretically be associated with several neural processes. First, they may be associated with BOLD signal related to oculomotor behavior in the neural oculomotor circuits, specifically in the frontal eyefields and intraparietal sulcus (Connolly et al., 2005). As the corrective oculomotor response after unaware errors is, however, only slightly different in terms of saccadic control and is occurring at high pace, fMRI signal may have failed to pick up these slight oculomotor differences on the small amount of trials. Second, following evidence from unconscious inhibitory control (van Gaal et al., 2010) a higher proportion of oculomotor adjustments after unaware errors as compared to aware errors may imply a higher level of unconscious inhibitory control. A higher level of inhibitory control has previously been associated with a higher level of activation in the inferior frontal cortex and the pre-supplementary motor area. In the current study the activation pattern for signal change on unaware errors as compared to aware errors did not show increased activation in the inferior frontal cortex, the pre-supplementary motor area, the frontal eyefields and the intraparietal sulcus. This suggests that no consistent and significant BOLD signal related to a higher proportion of behavioral adjustments after unaware errors was picked up, due perhaps to the too incidental and inconsistent occurrence and the slight oculomotor differences between corrected and uncorrected trials. This suggests that the contrast aware versus unaware errors should not be confounded by signal during unaware errors which is related to a higher proportion of behavioral adjustments after unaware errors.

### Anterior insula

The current results may provide information about the functional significance of AIC activation during error awareness. The plotted overlap of group-level activation patterns showed that the AIC responded to both error awareness and salience processing, while the PIC responded only to salience processing. Individual difference ROI analysis suggested that higher activation in AIC (but not in PIC) during salience processing predicted higher AIC activation to error awareness. In the individual difference analysis we however only observed a tendency toward higher correlations between error awareness and salience processing specifically in the AIC. The difference between the correlation coefficients of this significant correlation in the AIC on the one hand and the non-significant correlations on the other hand (between error awareness and salience processing in the PIC; and between error awareness and oddball processing *per se*) did not reach significance level. The results from the individual difference analysis in the AIC therefore lack specificity. The results of the ROI-based ANOVA-analyses of average regression weights indicated that within subjects the insula shows significant percent signal change in both error awareness and the processing of motivational significance (no significant main effect of task), but that the anterior insula is significantly more involved in both processes than the posterior insula (significant main effect of insular sub regions). Furthermore there is a tendency toward more AIC involvement and less PIC involvement in error awareness than in the processing of motivational significance in the oddball task.

In the light of previous findings on structural and functional connectivity of AIC, the currently observed similarity in AIC activation during error awareness and salience processing suggests that neural activity during both cognitive processes has direct access to similar larger scale neural systems. In contrast to the posterior part of the insula with few structural frontal projections, the AIC has been shown to be associated with strong frontal connectivity in studies of human probabilistic tractography. This anterior insula-frontal structural connectivity has been associated with the emotional salience and the cognitive control network linked to the implementation of goal-directed behavior (Cloutman et al., 2012). A recent investigation of insula-based resting-state fMRI has revealed similar results: whereas the posterior insula was functionally connected with primary and secondary somatomotor cortices; the dorsal anterior to middle insula was connected with dorsal ACC, along with other regions of the control network; and a ventral anterior region was primarily connected with pregenual ACC (Deen et al., 2011). Thus, error awareness and salience processing activate anterior subdivisions of the insula which seem ideally situated to communicate and integrate information within the salience network. This hypothesis might be effectively addressed by future studies combining structural and functional connectivity of AIC.

### Ventral versus dorsal anterior insula

The overlap of error awareness and the monitoring of motivationally significant events in the ventral AIC as visible in the plotted overlap of the two group-level data-sets, did not survive the conservative thresholds of the spatial overlap analysis in the “contrast masking analysis.” The contrast masking analysis however may have its methodological drawbacks, as the between-subject jitter in activation may prevent detecting activation overlap at the group-level, in particular in structures with a more distributed activation pattern of smaller voxel clusters. In the current study, it seems indeed that activation overlaps in larger and more continuous voxel clusters (such as thalamus and ACC) survived the “contrast masking” approach, whereas the smaller and more distributed voxel clusters in the insula may not have survived. While for the insula the contrast masking approach may have a drawback, the fact that contrast masking performed adequate on most of the structures involved at the whole-brain level, led us to include the contrast masking, approach into the paper. For the insula cortex, we have supplemented the contrast masking analyses with additional ROI analysis. The ROI analysis showed that the AIC is significantly involved in both task settings. The contrast masking analysis suggested further that error awareness activated predominantly the ventral AIC, whereas salience processing (the TTI effect) seemed to activate the AIC with maxima in the dorsal AIC. A functional dorsal-ventral distinction within anterior insula has not yet received much emphasis in the experimental literature on cognitive control, but has recently been addressed in a meta-analysis (Ullsperger et al., 2010). In a refined meta-analysis of 55 fMRI studies Ullsperger et al. focused on the patterns of co-activation of AIC and ACC across conditions that call for adjustments. They found that conditions of pre-response conflict (arising when a stimulus elicits competing response tendencies) and decision uncertainty (referring to situations when information about the correct response is underdetermined) primarily activated the dorsal part of AIC. Both conditions indicate an increased risk of imminent error, but the error might still be countermanded if the conflict is resolved or the uncertainty is reduced in time. By contrast, action slips and negative feedback cannot be repaired, but do call for remedial actions compensating the failure and/or subsequent adjustments improving future performance; these conditions predominantly activated the ventral part of AIC. Thus, the dorsal and ventral subregions of the AIC appear to play partially different roles in conditions that call for adjustments. The dorsal AIC appears to be involved in signaling increased risk (and hence the anticipation of imminent errors); the ventral AIC appears to register prediction error. Thus the dorsal AIC appears important in prospective control (recruiting the necessary effort to pre-empt potential risks and failures), whereas the ventral subdivision appears more important for reactive processing (monitoring for the need to undertake remedial action and homeostatic regulation; Lamm and Singer, 2010; Ullsperger et al., 2010). The current results seem to be in line with this proactive/reactive account of dorsal/ventral anterior insula.

Here, dorsal AIC activation during salience processing may reflect increased prospective control, due to the increased effort necessary to recruit sufficient resources to stay alert until the next target stimulus. In experimental research, the effect of a fore period on the reaction to a target stimulus has often been used as an independent variable of primary interest (Los et al., 2001). The focus of interest is the process of attaining and maintaining a state of potential action toward a future target event. Reaction time in reaction to a target stimulus, following a preparational period is commonly accepted as behavioral index for the efficiency of preparation (Jennings et al., 1998). A fast reaction should index that the participant is optimally ready to respond whereas a slow reaction would index that the participant is unprepared. Thus, on targets in a prepared state we should see low reaction times. Here, in the oddball task, reaction time after the three different TTIs did not differ significantly, suggesting that subjects attained a preparation state across longer intervals that sufficed to maintain reaction time. Functionally, the preparation state can take many different guises, ranging from the simple presetting of a motor response to complex cognitive preparation. Whatever form preparation takes, though, it is always oriented toward some goal, and takes time to reach a level that is optimal for that goal. The preparation process across trial-to-trial interval has been described to rely on the principle of “trace conditioning” (Los et al., 2001; Los and Schut, 2008). Trace conditioning refers to an inverted u shaped function describing a high preparatory state that is quickly attained but hard to maintain over time, wherein the participant aims at synchronizing the preparation peak and the imperative moment in order to produce a fast response. The most characteristic for the trace conditioning model of preparation is that the response preparation declines if its corresponding critical target occurs prior to the expected moment of the response, but remains unchanged if its critical target occurs after the expected moment (Los et al., 2001). In the current study the similar reaction times after all three interval conditions suggest that optimal preparation has been maintained across longer intervals until the expected (oddball) target occurred. In speculation, an initially increased and then maintained level of preparation aimed to rapidly respond to an anticipated stimulus may partly be reflected in the higher activation to targets after longer as compared to shorter TTIs with equal reaction speed. Hence, dorsal AIC activation during salience processing may reflect increased prospective control, due to the increased effort necessary to recruit sufficient resources to stay alert until the next target after a longer interval. This remains however speculative and can adequately be addressed by experimental paradigms that allow for measuring BOLD signal during the interval. The ventral AIC activation during error awareness in contrast may reflect reactive control due to the need to take remedial action.

Additionally, the increased ventral AIC activation during specifically error awareness (as compared to salience processing), may reflect physiological arousal related to an aversive affective response. Error awareness has been related to increases in peripheral physiological response (O’Connell et al., 2009; Wessel et al., 2011). Consistently, a recent meta-analysis found that peak coordinates from studies linking brain activation to peripheral physiological responses related to emotional experiences, such as heart-rate or galvanic skin response, tended to lie in ventral AIC (Mutschler et al., 2009).

Following this thought, the functional activation of ventral AIC during error awareness may also relate to the experienced valence of a salient event as an error is likely to be experienced as more unpleasant than a parametric oddball-target. Both dorsal and ventral AIC activation has been observed in response to unpleasant or disgusting odorants and aversive tastes (Zald et al., 1998; Wicker et al., 2003) and disgusting images (Calder et al., 2007). The ventral AIC, in particular, has been consistently found to be modulated by the hedonic valence of olfactory and gustatory stimuli (Royet et al., 2003). Ventral AIC activations to disgusting stimuli may reflect affective response to disgusting stimuli, while the dorsal AIC is involved in linking this affective response to attentional or executive mechanisms, similar to such divisions in pain processing (Baliki et al., 2009). The current results seem to support this functional affective/cognitive distinction of dorsal/ventral insula.

Another proposal is that the AIC contributes to the conscious error processing by generating a form of orienting response toward the error (Ullsperger et al., 2010). The current results partly encourage this proposal. The direct activation overlap in the dorsal ACC during error awareness and oddball processing might point to the generation of autonomic arousal processes in both tasks. As described above, the dorsal ACC has been consistently related to the generation of peripheral arousal. The AIC in turn has been related to the mapping of the arousal response (Critchley et al., 2005). The currently observed activation of the AIC during both error awareness and oddball processing may reflect the AIC mapping of the dorsal ACC arousal response. By mapping the arousal response the AIC may ascribe emotional significance to deviant targets and perceived errors and initiate the integration of the salient information into decision making processes to guide behavioral responses. In this context, errors may be homeostatically more salient and experienced emotionally as more aversive than a rare/deviant oddball-target. Thus, the activation in specifically the ventral AIC to aware errors might relate to increased peripheral arousal linked to an aversive affective response to the error. This aversive arousing component may be functional in the sense that it may increase the likelihood that the neural and peripheral system takes immediate remedial action.

A potentially informative next step for future research seems to be functional connectivity analysis of coordinated activity between ACC and ventral versus dorsal AIC during error awareness and oddball processing. As of yet, network research has not yet been able to consistently dissociate ventral versus dorsal AIC function based on its network profile in humans (Cloutman et al., 2012) In general agreement with insula patterns of structural connectivity in the macaque (Mesulam and Mufson, 1982a,b; Mufson and Mesulam, 1982) studies of human functional connectivity revealed ventral AIC to be correlated mostly with dorsal ACC, while dorsal and posterior insula correlated with more posterior parts of ACC (Deen et al., 2011). In humans however, in contrast to the consistency with which AIC–ACC functional connectivity has been identified using human resting-state measures, white matter connections between the two areas in the human brain have been failed to be demonstrated or only inconsistently observed via tractographic methods, if at all (van den Heuvel et al., 2009). Future studies combining measures of peripheral arousal with neural network analysis may show if the dorsal and ventral AIC form distinct pathways by which different aspects of salient neural signal, such as peripheral arousal or valence, can differentially mediate cognitive control and behavior.

## Conflict of Interest Statement

The authors declare that the research was conducted in the absence of any commercial or financial relationships that could be construed as a potential conflict of interest.

## Footnotes

^1^Subsample of Harsay et al. Shifts between default mode and task-focused brain networks during error awareness (*article submitted for publication*). To ensure a sufficient number of errors only participants with a minimum of 15 errors in the aware/unaware condition, with a false alarm rate lower than the aware/unaware error rates, and a maximum of 5% of self-rated uncertainty (based on a 1–100% post-experimental self-rating scale of uncertainty in performance evaluation) participated in the scanning session.

^2^www.datacolor.eu

^3^www.neurobs.com

^4^www.arringtonresearch.com

^5^www.mrivideo.com

^6^www.java.com

^7^www.fmrib.ox.ac.uk/fsl

^8^http://www.fmrib.ox.ac.uk/fls/

^9^http://www.spss.com

## Appendix

**Table A1:** **Complete list of brain regions showing significant BOLD activation during aware errors as compared to unaware errors**.

Brain region	*X*	*Y*	*Z*	Max *z*
R anterior insula cortex	34	18	−12	3.65
R mid insula cortex	50	8	−4	3.63
R postcentral gyrus (somatosensory cortex BA2R)	54	−26	44	3.04
L postcentral gyrus (somatosensory cortex BA2L, BA1L, BA3bL)	−46	−28	50	4.86
R thalamus	10	−24	10	3.15
L thalamus	−8	−22	8	4.39
R brain stem	8	−30	−8	3.21
R rostral anterior cingulate cortex	2	26	16	3.18
L rostral anterior cingulate cortex	−2	26	16	3.18
R dorsal anterior cingulate cortex	4	20	36	3.03
L dorsal anterior cingulate cortex	−4	32	32	3.64
R supplementary motor cortex (BA6R)	6	8	56	3.63
L supplementary motor cortex (BA6L)	−6	6	60	3.04
R precuneus cortex	4	−68	42	3.08
R inferior frontal gyrus	52	12	20	3.06
L inferior frontal gyrus	−48	12	22	2.48
R frontal eyefields BA8R, BA6R	20	−4	70	3.63
L frontal eyefields BA8L, BAL	−28	−26	70	4.22
R anterior intraparietal sulcus	−50	−44	50	4.23
L anterior intraparietal sulcus	40	−48	50	3.63
R parietal occipital junction (superior parietal lobe/lateral occipital lobe)	36	−58	40	3.72
L parietal occipital junction (superior parietal lobe/lateral occipital lobe)	−32	−60	40	4.30

*Aware errors >unaware errors, BOLD activation cluster-corrected at *z* = 2.3, *p* = 0.001. Coordinates are given in MNI space*.

**Table A2:** **Complete list of brain regions showing significant BOLD activation during target detection as a function of parametrically increasing interval length. during odd 3 as compared to odd 1**.

Brain region	*X*	*Y*	*Z*	Max *z*
R anterior insula cortex	34	14	−4	2.94
L anterior insula cortex	−40	16	−6	2.94
L mid insula cortex	−40	−12	10	2.82
L postcentral gyrus	−42	−34	50	3.09
R thalamus	14	−22	8	3.39
L thalamus	−10	−22	8	2.91
R brain stem	10	−22	−12	2.80
Supplementary motor area	2	−12	64	2.85
Dorsal anterior cingulate cortex	2	8	44	3.28
Supplementary motor area	2	−12	64	2.85
Precuneus cortex	2	−64	56	2.71
L dorsolateral prefrontal cortex	−38	50	20	2.67
R inferior frontal gyrus	52	12	20	3.06
L inferior frontal gyrus	−48	12	22	2.48
R premotor cortex (frontal eyefields BA8R, BA6R)	20	−4	70	3.63
L premotor cortex (frontal eyefields BA8L, BAL)	−28	−26	70	4.22
R anterior intraparietal sulcus	−50	−44	50	4.23
L anterior intraparietal sulcus	40	−48	50	3.63
R parietal occipital junction (superior parietal lobe/lateral occipital lobe)	36	−58	40	3.72
L parietal occipital junction (superior parietal lobe/lateral occipital lobe)	−32	−60	40	4.30

*Activation during oddball detection as a function of parametrically increasing interval length BOLD activation cluster-corrected at *z* = 2.3, *p* = 0.05. Coordinates are given in MNI space*.

**Table A3:** **Spatial overlap map of clusters of activation that survived, within each participant’s native space, both the threshold for the awareness contrast and the threshold for parametric TTI effects during target detection**.

Brain region	*X*	*Y*	*Z*	Max *z*
R thalamus	8	−26	0	2.89
L thalamus	−6	−26	4	3.25
R supplementary motor area	6	8	64	3.95
L supplementary motor area	−8	6	64	3.18
R dorsal ACC	2	18	26	2.39
L dorsal ACC	−8	34	24	3.21
Precunus	14	−64	46	2.82
L somatosensory cortex	−46	−32	46	3.56
R lateral occipital cortex	40	−64	44	3.59
L lateral occipital cortex	−38	−64	52	3.56

*Local maxima of activation of all significant clusters (at *z* = 2.3, *p* = 0.05, cluster-corrected) varying with aware errors and with the interval effect on target detection. All coordinates are given in MNI space*.
